# Diflunisal Attenuates Virulence Factor Gene Regulation and Phenotypes in *Staphylococcus aureus*

**DOI:** 10.3390/antibiotics12050902

**Published:** 2023-05-13

**Authors:** Liana C. Chan, Mihyun Park, Hong K. Lee, Siyang Chaili, Yan Q. Xiong, Arnold S. Bayer, Richard A. Proctor, Michael R. Yeaman

**Affiliations:** 1 Division of Molecular Medicine, Harbor-UCLA Medical Center, Torrance, CA 90502, USA; lchan@lundquist.org (L.C.C.); hlee@lundquist.org (H.K.L.); 2 Division of Infectious Diseases, Harbor-UCLA Medical Center, Torrance, CA 90502, USA; yxiong@lundquist.org (Y.Q.X.);; 3 Department of Medicine, David Geffen School of Medicine at UCLA, Los Angeles, CA 90024, USA; 4 Institute for Infection and Immunity, The Lundquist Institute for Biomedical Innovation at Harbor-UCLA Medical Center, Torrance, CA 90502, USA; 5 Vanderbilt Eye Institute, Vanderbilt University Medical Center, 2311 Pierce Ave., Nashville, TN 37232, USA; 6 Departments of Medical Microbiology/Immunology and Medicine, University of Wisconsin School of Medicine and Public Health, Madison, WI 53705, USA

**Keywords:** salicylates, diflunisal, virulence, antimicrobial, MRSA, *Staphylococcus aureus*

## Abstract

Virulence factor expression is integral to pathogenicity of *Staphylococcus aureus*. We previously demonstrated that aspirin, through its major metabolite, salicylic acid (SAL), modulates *S. aureus* virulence phenotypes in vitro and in vivo. We compared salicylate metabolites and a structural analogue for their ability to modulate *S. aureus* virulence factor expression and phenotypes: (i) acetylsalicylic acid (ASA, aspirin); (ii) ASA metabolites, salicylic acid (SAL), gentisic acid (GTA) and salicyluric acid (SUA); or (iii) diflunisal (DIF), a SAL structural analogue. None of these compounds altered the growth rate of any strain tested. ASA and its metabolites SAL, GTA and SUA moderately impaired hemolysis and proteolysis phenotypes in multiple *S. aureus* strain backgrounds and their respective deletion mutants. Only DIF significantly inhibited these virulence phenotypes in all strains. The kinetic profiles of ASA, SAL or DIF on expression of *hla* (alpha hemolysin), *sspA* (V8 protease) and their regulators (*sigB*, *sarA*, *agr* (RNAIII)) were assessed in two prototypic strain backgrounds: SH1000 (methicillin-sensitive *S. aureus*; MSSA) and LAC-USA300 (methicillin-resistant *S. aureus*; MRSA). DIF induced *sigB* expression which is coincident with the significant inhibition of RNAIII expression in both strains and precedes significant reductions in *hla* and *sspA* expression. The inhibited expression of these genes within 2 h resulted in the durable suppression of hemolysis and proteolysis phenotypes. These results indicate that DIF modulates the expression of key virulence factors in *S. aureus* via a coordinated impact on their relevant regulons and target effector genes. This strategy may hold opportunities to develop novel antivirulence strategies to address the ongoing challenge of antibiotic-resistant *S. aureus*.

## 1. Introduction

*Staphylococcus aureus* is an important human pathogen responsible for a broad range of infections causing significant morbidity and mortality worldwide [1,2,3,4,5]. The ability of *S. aureus* to cause myriad disease manifestations is mediated by the coordinated expression of an extensive repertoire of virulence factors, including exotoxins and proteolytic enzymes. Moreover, the rapid emergence of strains exhibiting multidrug resistance phenotypes (e.g., methicillin-resistant *S. aureus* (MRSA); vancomycin-intermediate *S. aureus* (VISA)) has accelerated the search for novel strategies to prevent or mitigate *S. aureus* infections. Therefore, the identification of molecules that interfere with virulence factor regulation and/or expression represents a potentially viable therapeutic strategy.

Salicylate compounds have previously been found to modulate *S. aureus* gene expression and virulence in vitro and in vivo [6,7,8,9,10,11]. Specifically, aspirin (acetyl-salicylic acid; ASA) and its primary metabolite, salicylic acid (SAL) appear to reduce the severity and progression of *S. aureus* infections in multiple clinical settings, including infective endocarditis (IE), hemodialysis-related tunnel catheter bacteremia, pacemaker- and other cardiac device-related infections and prosthetic joint infections [12,13,14,15,16]. Furthermore, ASA and SAL have demonstrated efficacy against MRSA in several experimental models of infection, including IE, bacteremia and osteomyelitis [10,11,12,17]. Our original studies [18,19,20,21,22], subsequently supported by other reports, suggested that such salicylates attenuate virulence through interactions with global regulatory systems [10,23,24]. 

**Figure 1:**
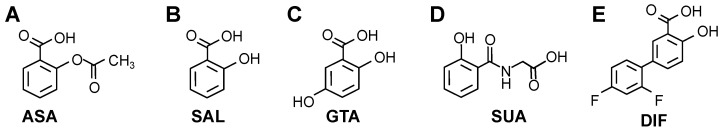
Chemical structure of study compounds. Study compounds included the parent compound ASA (aspirin; acetylsalicylic acid) (**A**), SAL (salicylic acid) (**B**), GTA (gentisic acid) (**C**) and SUA (salicyluric acid) (**D**) and the salicylate analogue DIF (diflunisal) (**E**).

In the current study, ASA and its major metabolites, SAL, gentisic acid (GTA) and salicyluric acid (SUA), as well as structural analogue, diflunisal (DIF) (Figure 1) were each assessed for their ability to inhibit virulence regulation and associated phenotypes in well-characterized *S. aureus* strains. Each of these metabolites is physiologically relevant, as nearly all ASAs are rapidly converted to SAL, GTA and SUA in vivo [25,26]. These compounds have been shown to exert beneficial anti-infective and anti-inflammatory properties in humans and experimental models of disease [27,28]. Similar to ASA, DIF is a non-steroidal anti-inflammatory drug (NSAID) that is frequently prescribed in clinical settings for the treatment of cardiac amyloidosis and arthritis [29,30]. In addition to its antivirulence effects on *S. aureus* in vitro, as well as in cutaneous and endovascular models of infection [10,11,18,19,20,21,22,24], DIF has been shown to reduce bone destruction during experimental *S. aureus* osteomyelitis [16]. Specifically, the impact of the above compounds on hemolysin and protease phenotypes, as well as on the kinetics of their respective regulatory (*sigB*, *agr*, *sarA*) and effector genes (*hla* (α-hemolysin), *sspA* (V8 protease)) were compared using a panel of strategic *S. aureus* strains (Table 1).

## 2. Results

### 2.1. Study Compounds Did Not Impede S. aureus Growth

**Figure 2:**
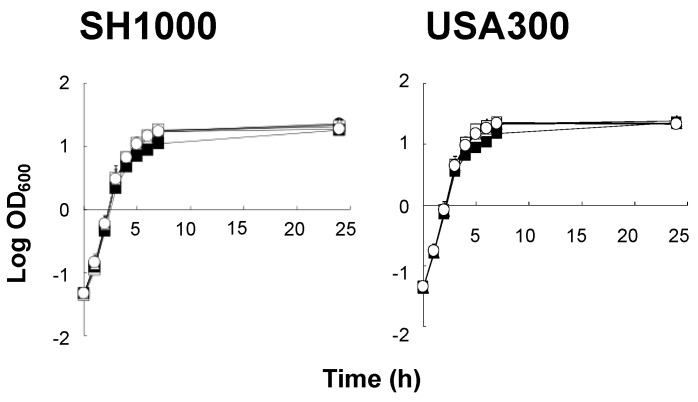
Study compounds do not affect growth of *S. aureus* strains. Growth curve analyses were performed to assess the direct anti-staphylococcal activity of test compounds. Log-phase organisms were inoculated into fresh BHI broth (OD_600_ = 0.05; inoculum 5 × 10^7^ CFU/mL) containing a given study compound (range: 0, 10, 25, 50, 100 μg/mL) and incubated at 37 °C with shaking. Bacterial growth was analyzed by spectrophotometry (OD_600_) at 1–8 and 24 h timepoints. (○ Control; ● ASA 25 μg/mL; △ SAL 25 μg/mL; ▲ GTA 25 μg/mL; □ SUA 25 μg/mL; ■ DIF 25 μg/mL).

The impact of the study compounds on the growth of SH1000 and LAC *S. aureus* strains in vitro was assessed. No observable growth impairment of either strain occurred over a 24 h time course (Figure 2). 

### 2.2. Study Compounds Differentially Modulated Hemolysin Activity in S. aureus

**Figure 3:**
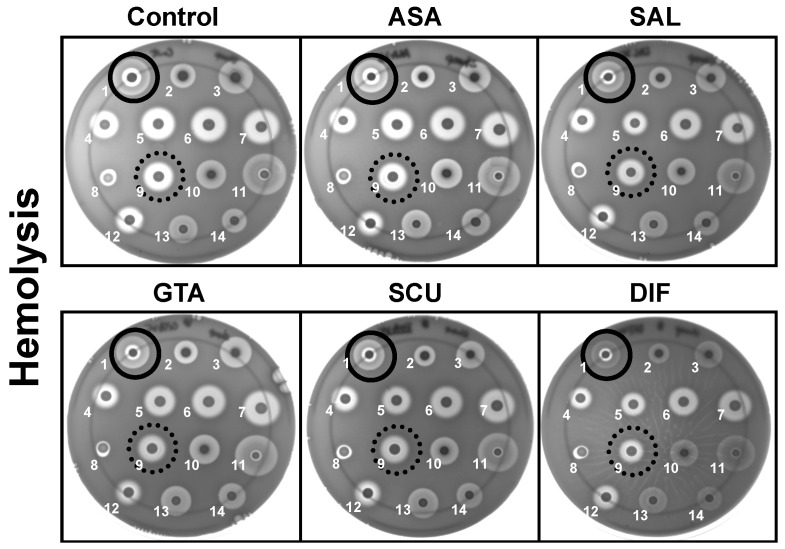
Study compounds affect hemolysis production of *S. aureus* strains. Log-phase organisms inoculated onto blood agar plates containing 25 μg/mL of ASA, SAL, GTA, SUA or DIF, or no compound. Plates were incubated for 24 h for bacterial growth followed by cold shock (4 °C, 4 h) for hemolysin activity. Zones of clearing were measured and normalized to no compound control. Closed circled colony (top left) is SH1000. Dotted circled colony (third row, second position) is LAC. Data are presented in Figure 4 as percent of control. Top row (left to right): SH1000 (1), SH1000 Δ*agr* (2), RN6390 (3); second row (left to right) FDA486 (wt) (4), FDA486 Δ*rsbU* (ALC2128) (5), FDA486 Δ*rsbV* (ALC2129) (6), FDA486 Δ*rsbW* (ALC2130) (7); third row (left to right) MW2 (8), LAC (9), ISP479R (10), ISP479C (11); fourth row (left to right) ATCC29213 (12), COL (13), COL Δ*agr* (14).

**Figure 4:**
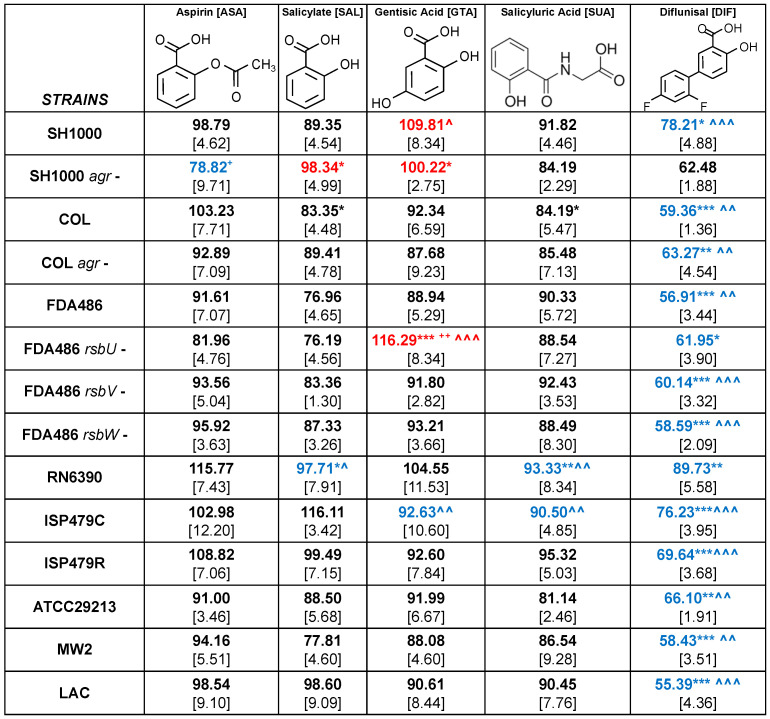
Relative hemolysis of *S. aureus* strains exposed to study compounds. Quantitative analyses of zones of clearing were measured and normalized to no compound control. Mean (bold) values and standard deviations (brackets) for each strain and compound combination are presented. Statistics were performed using two-way ANOVA and presented as: * *p* < 0.05, ** *p* < 0.01 and *** *p* < 0.001 for compound vs. ASA. ^ *p* < 0.05, ^^ *p* < 0.01 and ^^^ *p* < 0.001 for DIF vs. SAL. ^+^ *p* < 0.05 and ^++^ *p* < 0.01 for mutant vs. parent. Significantly decreased values are presented in blue while increased values are presented in red. * See statistical outcomes key in the Materials and Methods section for more details.

The impact of the study compounds on hemolytic phenotypes is summarized in Figure 3 and Figure 4. Overall, the study compounds exerted differential effects on hemolysis in the *S. aureus* study strain set. DIF exposures resulted in marked reductions in the percentage of hemolysis relative to control for all study strains as compared to ASA or any of its metabolites. This outcome was true regardless of genetic background (e.g., USA100 (COL), USA300 (LAC), USA400 (MW2)) or classical laboratory (RN6390) or reference strain (ATCC29213). As SAL is the primary biometabolite of ASA, compounds were compared to SAL in reducing hemolysis.

To explore the role of virulence factor regulation relative to compound efficacy, a panel of strategic mutants was evaluated. When comparing mutants against WT parents, only ASA yielded a greater inhibition of hemolysis in the SH1000 *agr*-deficient mutant (78.82 vs. 98.79; *p* = 0.05). However, this mutant had a significantly greater hemolysis in the presence of SAL or GTA (98.34 vs. 89.35; *p* = 0.02 and 100.22 vs. 109.81; *p* = 0.01, respectively) as compared to ASA (Figure 4). To explore the impact of the *sigB* regulon on hemolysis modulation by study compounds, *rsbU*-, *rsbV*- and *rsbW*-deficient mutants were studied in the FDA486 MSSA background (Figure 3 and Figure 4). Only GTA exposure revealed a significantly greater hemolysis in the FDA486 *rsbU*-deficient mutant as compared to its parent (116.29 vs. 88.94; *p* = 0.0017; Figure 4). No significant differences in the inhibitory effects of other study compounds were observed with respect to *rsb*-deficient mutants as compared to the parent. Likewise, SAL, GTA SUA and DIF exerted a greater modulation of hemolysis than SAL in RN6390 and ISP479C, but only DIF did so in the ISP479 *snoD* mutant. Overall, DIF exhibited a significantly greater inhibition of hemolysis relative to all other study compounds in the diverse panel of strains tested.

### 2.3. Study Compounds Differentially Inhibited Proteolysis Activity in S. aureus

**Figure 5:**
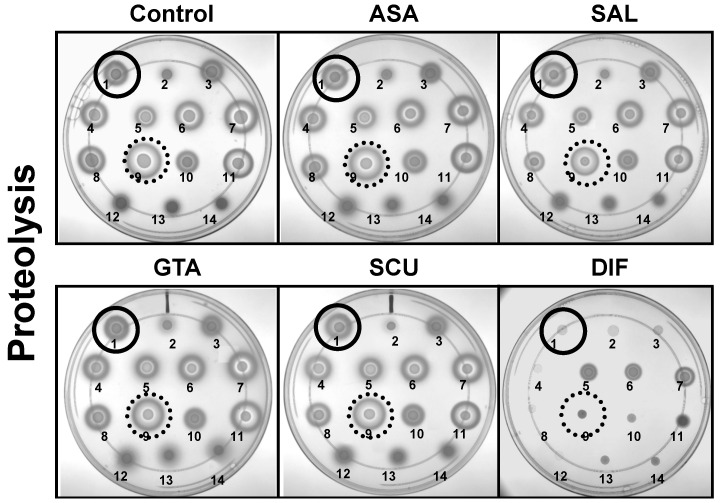
Study compounds affect proteolysis production of *S. aureus* strains. Log-phase organisms were inoculated onto standard-method caseinate agar plates containing 25 μg/mL of ASA, SAL, GTA, SUA or DIF, or no compound. Plates were incubated for 24 h for bacterial growth followed by cold shock (4 °C, 4 h) for hemolysin activity. Zones of clearing were measured and normalized to no compound control. Closed circled colony (top left) is SH1000. Dotted circled colony (third row, second position) is LAC. Data are presented in Figure 6 as percent of control. Top row (left to right): SH1000 (1), SH1000 Δ*agr* (2), RN6390 (3); second row (left to right) FDA486(wt) (4), FDA486 Δ*rsbU* (ALC2128) (5), FDA486 Δ*rsbV* (ALC2129) (6), FDA486 Δ*rsbW* (ALC2130) (7); third row (left to right) MW2 (8), LAC (9), ISP479R (10), ISP479C (11); fourth row (left to right) ATCC29213 (12), COL (13), COL Δ*agr* (14).

**Figure 6:**
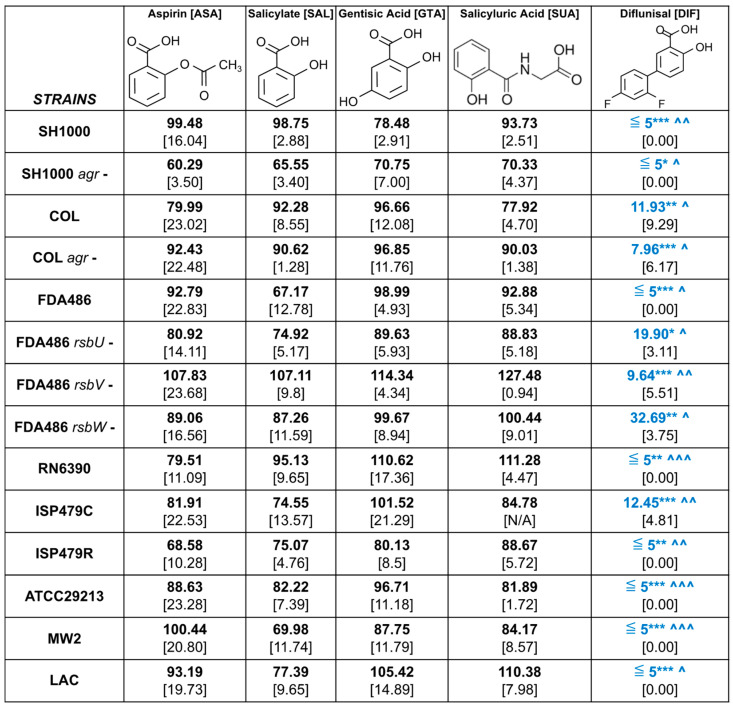
Relative proteolysis of *S. aureus* strains exposed to study compounds. Quantitative analyses of zones of clearing were measured and normalized to no compound control. Mean (bold) values and standard deviations (brackets) for each strain and compound combination are presented. Statistics were performed using two-way ANOVA and presented as: * *p* < 0.05, ** *p* < 0.01 and *** *p* < 0.001 for compound vs. ASA; ^ *p* < 0.05, ^^ *p* < 0.01 and ^^^ *p* < 0.001 for DIF vs. SAL. Significantly decreased values are presented in blue while increased values are presented in red. * See statistical outcomes key in the Materials and Methods section for more details.

The impacts of ASA, its metabolites or DIF on proteolytic phenotypes are summarized in Figure 5 and Figure 6. Only DIF exerted significant reductions in the percentage of proteolysis relative to control for all study strains. Moreover, DIF achieved a significantly greater inhibition of proteolysis than ASA in all study strains. Relative to SAL, only DIF exhibited a significantly greater inhibition of proteolysis in most strains studied. No significant differences were observed in terms of DIF inhibition of proteolysis in any parent vs. mutant strain pairs.

In comparison to hemolysis, the components of the *rsb* regulon had significant differences relative to their impact on DIF efficacy in suppressing proteolysis (Figure 5 and Figure 6). For example, as compared to the parent, proteolysis in *rsbU*- and *rsbW*-deletion mutants was significantly less inhibited by DIF (19.90 vs. ≤ 5; *p* = 0.006 and 32.69 vs. ≤ 5; *p* = 0.001, respectively). However, relative to the *rsbW*-deletion mutant, DIF exerted a significantly greater inhibition of proteolysis in the *rsbU*- and *rsbV*-deletion mutants (19.90 vs. 32.69; *p* = 0.029 and 9.64 vs. 32.69; *p* = 0.03, respectively). Together, these data suggest that the *rsb* regulon contributes to DIF efficacy in modulating proteolysis inhibition.

### 2.4. ASA, SAL and DIF Exhibited Differential Kinetics of Virulence Gene Inhibition

**Figure 7:**
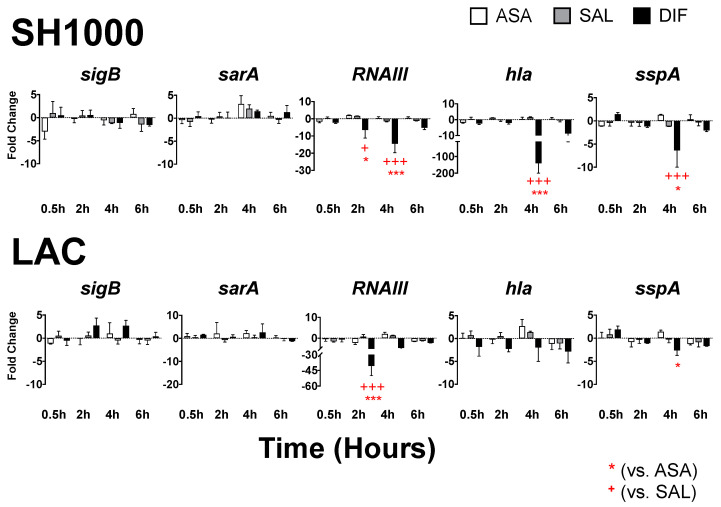
DIF inhibited virulence factor mRNA expression. Expression of regulatory genes *RNAIII* (*agr*), *sarA*, *sigB* as well as the predominant hemolysin *hla* (α–hemolysin) and protease *sspA* (V8 protease) genes were quantified over the course of 6 h, following exposure to ASA, SAL or DIF (25 μg/mL) as compared to control. DIF inhibited mRNA expression of *RNAIII, hla* and *sspA* as compared to ASA (* = *p* < 0.05; *** = *p* < 0.001) or SAL (^+^ = *p* < 0.05; ^+++^ = *p* < 0.001) in SH1000 (MSSA) or LAC (MRSA USA300).

To explore the mechanistic basis of study compounds which inhibited virulence phenotypes, we assessed the transcriptional kinetics of target structural and regulatory genes involved in hemolysis and proteolysis. Transcriptional profiles for two prototype strains (SH1000; and LAC-USA300) are summarized in Figure 7.

The compounds ASA, SAL and DIF were the focus of transcriptomic studies as these agents were phenotypically the most impactful on hemolysis and proteolysis. These compounds had differential effects on the quantity and kinetics of regulatory gene transcription in these two *S. aureus* strains (Figure 7; Supplementary Figure S1).

DIF significantly reduced *RNAIII* transcription within 2 h in both strains. The peak inhibition of *RNAIII* transcription occurred at 4 h for SH1000 (15-fold reduction; *p* = 0.001 vs. 0.5-h) and at 2 h for LAC (40-fold reduction; *p* < 0.0001 vs. 0.5-h). Consistent with this effect, the *RNAIII* counter-regulatory genes *sarA* or *sigB* increased in their expression in SH1000 or LAC, respectively. The *sarA* peak transcription occurred at 4 h post-exposure to ASA in SH1000 (*p* = 0.06 vs. 0.5-h). The *sigB* peak transcription occurred by 2 h post-exposure to DIF in LAC (*p* = 0.15 vs. 0.5 h). Study compounds did not significantly differ in their impact on *sarA* or *sigB* expression. Interestingly, *sarA* transcription did not appreciably increase in LAC, and *sigB* transcription did not appreciably increase in SH1000, regardless of time or compound. Notably, regulatory gene transcription essentially returned to baseline in both strains by 6 h post-exposure regardless of the study compound. 

Study compounds also had differential effects on the quantity and kinetics of virulence gene transcription in comparative *S. aureus* strains (Figure 7). Consistent with downregulated *RNAIII* transcription, in *SH1000*, DIF caused a significant reduction in *hla* (~140-fold) transcription at 4 h as compared to baseline; this effect was significantly greater than ASA or SAL (Figure 7). The reduction in *hla* transcription by DIF was observed at every time point in LAC, but did not reach significance relative to baseline or other compounds. In both strains, DIF inhibition of *sspA* expression was significant at 4 h post-exposure as compared to ASA or SAL (Figure 7). In SH1000, *hla* expression remained highly suppressed by DIF at 6 h, but did not achieve significance as compared to baseline or other study compounds. Likewise, DIF inhibition of *sspA* expression trended to return to baseline by 6 h in SH1000. In contrast, DIF inhibition of *sspA* expression in LAC continued to remain significant over the 6 h study period as compared to baseline (*p* = 0.01 vs. 0.5-h), but was only significant at 4 h as compared to ASA (Figure 7).

## 3. Discussion

Recent serendipitous clinical observations revealed that ASA exerts beneficial anti-infective efficacy in multiple human infectious diseases. For example, low-dose ASA and its de-acetylated metabolite SAL significantly decrease the risk of *S. aureus* bacteremia in patients with hemodialysis tunneled catheters and with infected prosthetic joints [12,13]. Experimental models also show that these compounds reduce the severity and progression of infective endocarditis [10,11,17]. These observations have prompted increasing interest in the potential to repurpose ASA or other nonsteroidal anti-inflammatory drugs (NSAIDs) as novel adjunctive anti-infective therapies. 

In the current study, the impact of ASA, its salicylate metabolites (SAL, GTA, SUA) and the fluorinated structural analogue, DIF, were studied for their modulation of virulence phenotypes and transcriptional regulation using a panel of defined *S. aureus* strains in vitro. ASA, SAL and DIF exerted inhibitory effects on hemolysis and proteolysis capabilities. However, only DIF inhibited both virulence phenotypes in all study strains, and did so significantly greater than ASA and SAL (Figure 4 and Figure 6). Interestingly, in SH1000 lacking *agr*, a greater inhibition of hemolysis was observed by ASA as compared to the wild-type parent strain. In contrast, *agr* did not significantly impact DIF efficacy in either SH1000 or COL backgrounds (Figure 4). Similarly, the presence or absence of *agr* did not affect proteolysis inhibition by DIF or other study compounds in all genetic backgrounds (Figure 6). These findings suggest *agr* alone is not a primary mechanism of DIF inhibition of hemolysis or proteolysis activity in *S. aureus*. The impact of genes within the *sigB* locus (*rsbU*, *rsbV*, *rsbW*) on study compound efficacy was also investigated. The deletion of *rsbV* or *rsbW* had no significant effect on hemolysis or proteolysis as compared to wild-type FDA486. However, the absence of *rsbU* (considered to be the “sensor” of this operon) [33] promoted hemolysis in the presence of GTA as compared to the parent. By comparison, the deletion of any of the *rsb* genes consistently reduced the efficacy of DIF proteolysis inhibition; none of these differences achieved statistical significance as compared to control. These findings suggest that *sigB* and its *rsb* components are involved directly or indirectly in the inhibitory mechanisms of DIF against proteolysis (Figure 6).

Next, we explored the transcriptional kinetics of target regulatory and effector genes in response to compounds ASA, SAL or DIF that were shown to inhibit virulence phenotypes. Gene expression was monitored over a 6 h period in prototypic MRSA and MSSA strains to enable the temporal assessment of transcriptional profiles in response to compound exposure. In prototypic MSSA and MRSA strains, distinct kinetic patterns of transcription were identified in response to specific study compounds. In SH1000 (MSSA), an increased expression of *sarA* by 4 h post-DIF exposure coincided with a significant suppression of *RNAIII*. By comparison, in LAC (MRSA), an increased expression of *sigB* by 2 or 4 h post-DIF exposure paralleled with the significant inhibition of *RNAIII*. These findings are consistent with *sigB* as a strong counter regulator of *RNAIII* [38,39]. Regardless of *sarA* or *sigB* regulation, suppression of *RNAIII* was strongly correlated with the inhibition of *hla* and *sspA*. These transcriptional profiles were concordant with the significant inhibition of hemolysis and proteolysis by DIF. It is notable that rapid transcriptional inhibition of *hla* and *sspA* by 2–4 h resulted in a durable suppression of their respective virulence phenotypes even at 24 h. Given that gene expression had largely returned to baseline by 6 h, early and/or temporary interference in virulence gene expression can have lasting effects that may benefit antivirulence efficacy. Neither ASA nor SAL significantly altered virulence gene expression in these strains over 6 h.

We recognize several potential limitations with our investigation. First, these in vitro outcomes may not fully represent *S. aureus* virulence regulation within the host. Preliminary data not presented here, support the efficacy of DIF in antibiotic therapy of MRSA infection in vivo [24]. Second, study compounds appeared to have relatively different degrees of activity against different *S. aureus* strains. However, the fact that DIF strongly inhibited *RNAIII*, *hla* and *sspA* expression, as well as hemolysis and proteolysis phenotypes in every studied strain is promising as a therapeutic strategy regardless of *S. aureus* genetic background. Third, the observation that hemolysis was not abolished in *agr*-deleted SH1000 and COL backgrounds suggests *agr*-independent regulation of *hla* and *sspA*. This unexpected finding has also been reported by Liu et al. [23,40] and suggests that novel anti-infective targets of virulence inhibition are yet to be explored.

**Figure 8:**
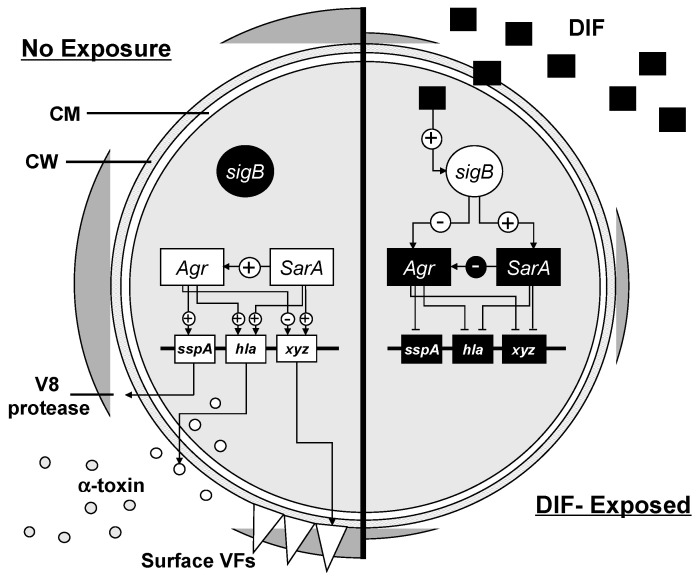
Hypothetical model of DIF–induced effects on regulatory and virulence gene expression in *S. aureus*. We hypothesize that DIF acts on *sigB* to inhibit *agr* and/or activate *sarA* gene expression. The downstream consequence of this regulatory effect is the repression of *sspA* and *hla* as well as other unknown targets (denoted as gene xyz). These latter genes are the subject of ongoing investigations.

The currents studies further substantiate our original findings regarding the antivirulence properties of ASA, its metabolites and the structural analogue DIF [20,21,22,24]. A hypothetical model integrating the putative mechanisms of DIF is illustrated in Figure 8. Our original observations have also been supported by work from several other laboratories [16,41,42,43]. In light of the burgeoning threat of MRSA resistance to multiple antibiotic classes, novel approaches to attenuate virulence without inducing resistance are attractive strategies. For example, the complementary inhibition of virulence factor expression and targets of conventional antibiotics may translate to a greater microbicidal impact, reduced emergence of resistance and enhanced immune-mediated efficacy in MRSA infection. The current findings provide further proof of concept that DIF and the structural analogues of SAL attenuate prototypic virulence factor expression in *S. aureus*. The translation of these strategies is currently under investigation in our laboratories. 

## 4. Materials and Methods

In this study, the potential for ASA and its relevant metabolites and DIF to modulate virulence factor expression in *S. aureus* was investigated. Study compounds included the parent ASA and metabolites, SAL, SUA and GTA; the salicylate analogue DIF was tested in parallel (diflunisal) (Figure 1).

### 4.1. Compounds

Unless otherwise noted, the following compounds were obtained from Sigma-Aldrich, Inc. (St. Louis, MO, USA) and prepared as stock solutions from powder: ASA; SAL; SUA (Acros Organics, NJ, USA); GTA; and DIF (Figure 1). Stock solutions were prepared in ethanol and stored at 4 °C until use. 

### 4.2. Bacterial Strains

*Staphylococcus aureus* strains (MSSA; and MRSA) used in this study are described in Table 1. Strains included both prototypic laboratory and well-characterized clinical isolates with known genotypes and phenotypes. For experiments detailed below, all strains were cultured to mid-logarithmic or stationary phase in brain–heart infusion broth (BHI; Difco Laboratories, Detroit, MI, USA), at 37 °C with agitation, washed, and resuspended in phosphate-buffered saline (PBS; Irvine Scientific, Santa Ana, CA, USA; pH 7.2). Experimental inocula were determined by spectrophotometry and validated by quantitative culture. 

### 4.3. Anti-Staphylococcal Activity of Compounds

Growth curve analyses were performed to assess the direct anti-staphylococcal activity of all test compounds. To do so, log-phase organisms were inoculated into fresh BHI broth (OD_600_ = 0.05; inoculum 5 × 10^7^ CFU/mL) containing a given study compound (range: 0, 10, 25, 50, 100 μg/mL) and incubated at 37 °C with shaking. At selected time-points (1–8 h and 24 h), cultures were analyzed by spectrophotometry (OD_600_) and quantitative culture in comparison to respective untreated controls (Figure 1). 

### 4.4. Influence of Compounds on Hemolysin or Protease Expression

Two pivotal phenotypes in *S. aureus* that govern many of its virulence capacities involve secretion of hemolysins and proteases [44]. The effects of our study compounds on expression of secreted hemolysins and proteases were compared in the panel of *S. aureus* strains summarized in Table 1. In these assays, blood agar-tryptic soy agar plates (TSA; Beckton Dickinson, CA) contained either 5% sheep or 5% rabbit blood (Hardy Diagnostics, CA) and 25 μg/mL of ASA, SAL, GTA, SUA or DIF, or no compound. These concentrations encompassed the known human blood levels for ASA and DIF after standard dose regimens [45]. Likewise, for protease assays, standard-method caseinate agar (SMCA) plates containing 25 μg/mL of each study compound were compared to control plates without these compounds. For these assays, strain inocula were prepared as above. To ensure that maximal hemolysin activity was detected, hemolysin assay plates were cold-shocked (4 °C for 4 h) prior to reading zones of hemolysis (α-hemolysin activity facilitated by temperature shock [46]). Zones of hemolytic or proteolytic activity were measured by quantitative imaging (AlphaEaseFC imager and software; Alpha Innotec, Kasendorf, Bayern, Germany). Statistics were performed using two-way ANOVA. Data are presented as: * *p* < 0.05, ** *p* < 0.01 and *** *p* < 0.001 for compound vs. ASA. ^ *p* < 0.05, ^^ *p* < 0.01 and ^^^ *p* < 0.001 for DIF vs. SAL. ^+^ *p* < 0.05 and ^++^ *p* < 0.01 for mutant vs. parent. 

### 4.5. Influence of Compounds on Gene Expression

To identify the influence of the study compounds on transcriptional correlates of the above two virulence phenotypes, expression of selected virulence regulon or effector genes were compared in prototypic MSSA (SH1000) and MRSA (LAC) strains. Expressions of regulatory genes *RNAIII* (*agr*), *sarA* and *sigB*, as well as the predominant hemolysin gene, *hla* (producing α-hemolysin) and the protease gene, *sspA* (producing V8 protease) were quantified over a 6 h time course, following exposure to ASA, or its analogues (vs. untreated controls). In brief, 10^9^ CFU of log phase cells were isolated and exposed for 1 h to ASA, SAL, GTA, SUA or DIF (concentration, 25 μg/mL). Control samples were exposed to buffer alone in the absence of the compound. In parallel experiments, organisms were cultured for 2 h, 4 h or 6 h in a fresh medium, and then exposed to these compounds as above. The expression of target genes-of-interest (Supplemental Table 1) as assessed by quantitative real-time PCR (qRT–PCR). In brief, mRNA was extracted from cells treated as above using standard methods, and purified using RNeasy (QIAGEN Inc., Germantown, MD, USA) and Turbo DNA-free (Ambion, Austin, TX, USA) kits per manufacturer’s instructions. Resulting mRNA was converted to cDNA using the RETROscript kit for reverse transcriptase PCR (RT-PCR; Ambion) per manufacturer’s instructions. Each cDNA template was then used for qRT-PCR based on target gene primers and optimized for ABI 7000 system implementing a SYBR green PCR master mix (Applied Biosystems, Foster City, CA, USA). In all cases, the threshold cycle (Ct) values were normalized to 16S rRNA. Relative fold changes in gene expression were determined using the 2^−ΔΔCt^ method.

## Figures and Tables

**Table 1:** *Staphylococcus aureus* strains used in this study.

Strain	Description	Reference
SH1000	Laboratory strain: 8325-4 with repaired *rsbU* mutation	American Type Culture Collection
SH1000 *agr*-	*agr*-null mutant of SH1000	[31]
COL	Original MRSA strain	American Type Culture Collection
COL *agr*-	*agr*-null mutant of COL	[32]
FDA486	Prototypic MRSA with intact *rsbU*	[33]
FDA486 *rsbU*-	*rsbU*-null mutant of FDA486	[34]
FDA486 *rsbV*-	*rsbV*-null mutant of FDA486	[34]
FDA486 *rsbW*-	*rsbW*-null mutant of FDA486	[34]
RN6390	8325-4 derivative with 11-bp deletion in *rsbU*	[33]
ISP479C	Plasmid-cured derivative of ISP479 (derived from 8325-4)	[35]
ISP479R	*snoD* mutant of ISP479C	[23]
ATCC29213	Laboratory reference strain	American Type Culture Collection
MW2	CA-MRSA USA400	[36]
LAC	CA-MRSA USA300 isolated from Los Angeles County Jail	[37]

## Data Availability

Data are contained within the article (Figure 2, Figure 4, Figure 6 and Figure 7). Additional datasets are available upon request.

## Supplementary Materials

The following supporting information can be downloaded at: https://www.mdpi.com/article/10.3390/antibiotics12050902/s1, Table S1: Quantitative heatmap of SAL and DIF impact on MRSA and MSSA virulence gene mRNA expression. Expression of regulatory (REG) genes *RNAIII* (*agr*), *sarA*, *sigB* as well as the predominant virulence factors (VIR) hemolysin *hla* (α-hemolysin) and protease *sspA* (V8 protease) genes were quantified over the course of 6 h following exposure to SAL or DIF (25 μg/mL) as compared to control. DIF inhibited the expression of *RNAIII*, *hla* and *sspA* as compared to SAL in SH1000 (MSSA) and USA300 (MRSA) backgrounds.
