# Supplementary figures and images for: Diflunisal Attenuates Virulence Factor Gene Regulation and Phenotypes in Staphylococcus aureus
Source: Antibiotics (Basel). 2023 May 13;12(5):902. doi: 10.3390/antibiotics12050902 (PMC10215304; doi:10.3390/antibiotics12050902)

Supplemental Figure S1

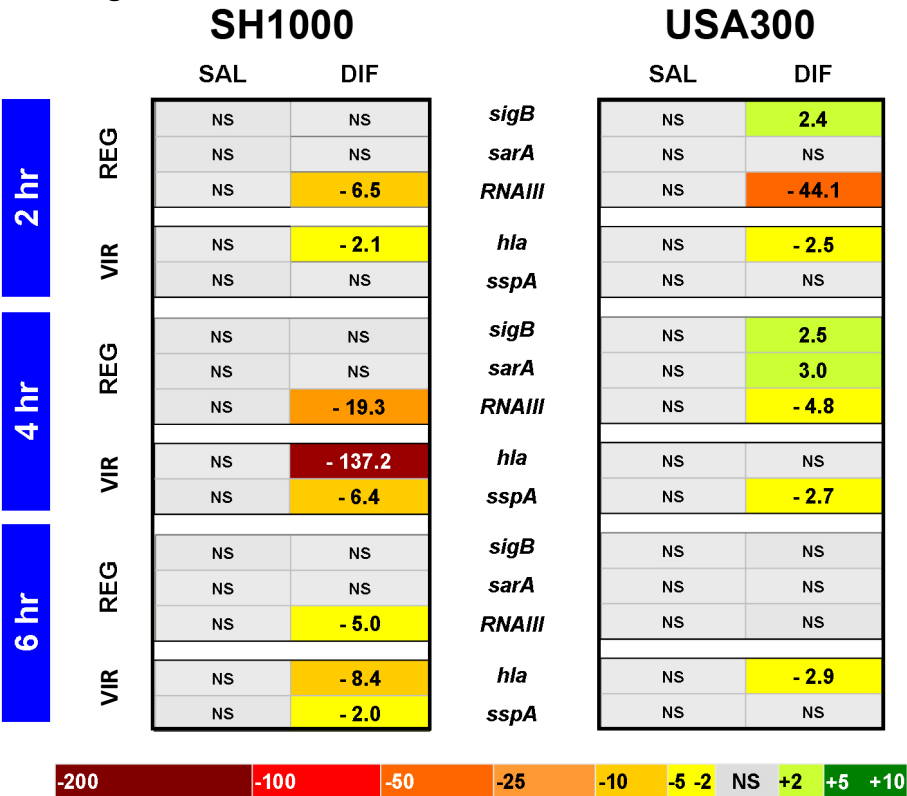

Supplement: Supplementary file 1 [file antibiotics-12-00902-s001.zip › antibiotics-2357073-supplementary.pdf]
